# *Lactobacillus casei* Strain Shirota Alleviates Constipation in Adults by Increasing the Pipecolinic Acid Level in the Gut

**DOI:** 10.3389/fmicb.2019.00324

**Published:** 2019-02-21

**Authors:** Yangwenshan Ou, Shanbin Chen, Fazheng Ren, Ming Zhang, Shaoyang Ge, Huiyuan Guo, Hao Zhang, Liang Zhao

**Affiliations:** ^1^Beijing Advanced Innovation Center for Food Nutrition and Human Health, College of Food Science and Nutritional Engineering, China Agricultural University, Beijing, China; ^2^Key Laboratory of Functional Dairy, College of Food Science and Nutritional Engineering, China Agricultural University, Beijing, China; ^3^Beijing Laboratory for Food Quality and Safety, China Agricultural University, Beijing, China; ^4^School of Food and Chemical Engineering, Beijing Technology and Business University, Beijing, China; ^5^Beijing Higher Institution Engineering Research Center of Animal Product, College of Food Science and Nutritional Engineering, China Agricultural University, Beijing, China; ^6^Hebei Engineering Research Center of Animal Product, Sanhe, China

**Keywords:** *Lactobacillus casei* strain Shirota, constipation, metabolomics, gut metabolites, pipecolinic acid

## Abstract

The benefits of probiotics for constipation are widely accepted, but the mechanisms involving gut metabolites are unclear. In this study, we investigated the effects of *Lactobacillus casei* strain Shirota (LcS) on constipated patients and revealed that a metabolite mediator is involved in the LcS-induced constipation alleviation. Sixteen constipated patients and 22 non-constipated participants were recruited. The subjects consumed 100 mL of an LcS beverage (10^8^ CFU/mL) per day for 28 days. The fecal non-volatile metabolites were determined by GC/MS, and the targeted metabolites were further verified in a constipated mouse model. In constipated patients, LcS intervention significantly improved defecation frequency (from 4.81 to 7.81 times per week, *p* < 0.05), stool consistency (from 2.52 to 3.68, *p* < 0.05) and constipation-related symptoms. A total of 14 non-volatile fecal metabolites were obtained as potential constipation-related metabolites that were regulated by LcS. Among these metabolites, pipecolinic acid (PIPA) had a significant positive correlation with defecation frequency in constipated patients. PIPA significantly promoted the small intestinal propulsive rate (from 25.45 to 39.68%) and increased the number of fecal pellets (from 30.38 to 57.38 pellets) in constipated mice (*p* < 0.05). The 5-hydroxytryptamine (5-HT) and acetylcholine (ACh) in colonic tissue may be partly involved in PIPA-mediated constipation alleviation. In conclusion, PIPA was a metabolic mediator in the gut that participated in LcS-induced constipation alleviation.

## Introduction

Constipation is defined as a difficulty in evacuating, which is characterized by a reduced defecation frequency (Bharucha, 2007). As a common disease, constipation impacts approximately 15% of the people in the world (Higgins and Johanson, 2004; Peppas et al., 2008; Mugie et al., 2011; Suares and Ford, 2011; Chu et al., 2014).

Many investigations have shown that probiotics could attenuate constipation in both animal model and human trials (Dimidi et al., 2014a,b; Qian et al., 2015; Wang L.L. et al., 2017). Different probiotic strains or mixed formulas not only reduced the symptoms of constipation but also alleviated constipation-related diseases, such as irritable bowel syndrome (IBS) and depressive behaviors (Xu et al., 2018). Koebnick et al. (2003) found that 2 weeks of consecutive ingestion of 65 mL/day of beverages containing 6.5 × 10^9^ CFU/mL *Lactobacillus casei* strain Shirota (LcS) led to a significant decline in the occurrence of hard and lumpy stool in constipated adults. *Bifidobacterium lactis* DN-173 010 at a dose of 1.25 × 10^10^ CFU for consecutive 4 weeks significantly reduced the colonic transit time by an average 12.2 h in constipated women (Agrawal et al., 2009). *Lactobacillus reuteri* DSM 17938 at a dose of 1.0 × 10^8^ CFU noticeably improved bowel movements by 2.6 per week in constipated adults after 4 weeks of treatment (Ojetti et al., 2014), and it also led to a significant alleviation of abdominal discomfort, pain and bloating in constipated adults after 105 days of administration (Riezzo et al., 2018).

Accumulating evidence suggests that bacteria-related gut metabolites possibly have an important role in constipation. Short chain fatty acids (SCFAs) are mainly produced from carbohydrates fermentation in the colon, and they contribute to the health of the human body (Wong et al., 2006). For constipation, decreased levels of SCFAs have been shown to have a negative association with prolonged transit time (Lewis and Heaton, 1997). Furthermore, it has also been found recently that the precursors of protein metabolism products, such as tryptophan and 5-hydroxytryptophan (5-HTP), were significantly negatively linked to a prolonged colonic transit time (Roager et al., 2016) and that the intraluminal release of 5-hydroxytryptamine (5-HT) could be stimulated by SCFAs (Fukumoto et al., 2003). Other protein catabolism products including urinary *p*-cresol-sulfate and *p*-cresol-glucuronide were shown to have a significant positive association with colonic transit time (Roager et al., 2016). In addition to the decline of defecation frequency and the increase in intestinal transit time, constipation is generally accompanied with hard stool and a low fecal water content. It has been shown that bile acids have opposite effects on water absorption in gut, which could cause water absorption at low concentration and lead to water secretion at high level (Russell et al., 1973). Gut bacteria were considered to be the regulators of these metabolites. Commensal intestinal microbiota including *Ruminococcus* and *Faecalibacterium* are the main butyrate producers in the gut, and probiotics such as *Bifidobacterium longum* SP 07/3, *Bifidobacterium bifidum* MF 20/5, *Lactobacillus acidophilus* CRL 1014 and *Lactobacillus rhamnosus* GG (LGG) have also been shown to generate SCFAs (LeBlanc et al., 2017). VSL#3, which is another commercial probiotic mixture, had the ability to enhance bile acids deconjugation and fecal excretion (Degirolamo et al., 2014). *Faecalibacterium prausnitzii*, which is an anti-inflammatory commensal bacterium in the human gut (Sokol et al., 2008), was negatively correlated with the protein metabolites *p*-cresol-sulfate and *p*-cresol-glucuronide (Roager et al., 2016), and probiotics such as LcS and *Bifidobacterium breve* also led to a significant decrease in *p*-cresol in healthy adults (De Preter et al., 2007). For 5-HT, its transporter expression has been noted to be upregulated in intestinal epithelial cells after the administration of an LGG supernatant (Wang et al., 2015). Although probiotics could regulate bacteria and metabolism in gut, studies rarely directly show how probiotics can alleviate constipation by regulating intestinal metabolites.

*Lactobacillus casei* strain Shirota is known for constipation alleviation in different populations (Koebnick et al., 2003; Sakai et al., 2011; Mazlyn et al., 2013; Aoki et al., 2014). Especially in previous random placebo-controlled double-blinded trails, LcS could improve defecation and alleviate constipation symptoms (Koebnick et al., 2003; Mazlyn et al., 2013). However, there is no adequate evidence to explain its mechanism on constipation alleviation. In this study, the constipation alleviating effects of LcS were evaluated in constipated adults, and the intestinal metabolites involved in LcS-induced constipation alleviation were investigated by metabolomic approaches. The potential constipation alleviating metabolites were further verified using a constipation mouse model. This study could add to the knowledge on the probiotic-induced mechanisms that alleviate constipation.

## Materials and Methods

### Study Subjects

The human study was carried out in accordance with the recommendations of Ethics Review on Biomedical Research Involving Human Subjects, National Health Commission of the People’s Republic of China, with written informed consent from all subjects. All subjects gave written informed consent in accordance with the Declaration of Helsinki. The protocol was approved by the ethics committee of People’s Liberation Army Beijing Military General Hospital (Research Project Identification No. 2015/105). Two groups of volunteers (constipated and non-constipated) were recruited from Beijing, China and were 18–55 years old. The constipated volunteers were screened according to the ROME III criteria (Sandler and Drossman, 1987; Longstreth et al., 2006). The non-constipated volunteers were defined by the following criteria: (A) defecation frequency > 3 times in the previous week; (B) no straining, sensation of incomplete evacuation, anorectal obstruction, extra aid needed to defecate and diarrhea when defecating in the previous week; and (C) defecation criteria conform to A and B for the past 3 months. Patients who used antibiotics, antimycotics, antidiarrheal medication or laxatives within 30 days of the study were excluded. Participants who were allergic to milk protein were also eliminated, as well as the patients who had pathogenesis rooted in organic or neurological lesions. Patients who took part in other similar studies within the 2 months before the study began were also excluded.

### Study Design

**FIGURE 1:**
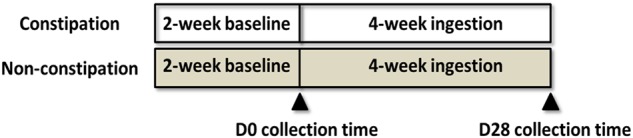
Human study design.

This open trial was 6 weeks long and included a 2-week baseline period and 4-week ingestion period (Figure 1). During the ingestion period, after their daily lunch, all subjects consumed one bottle (100 mL) of commercial fermented dairy beverage containing 1 × 10^8^/mL living LcS cells, obtained from the market. Laxatives, antibiotics, medications or other type of fermented dairy products were not allowed in the study. Fecal samples were collected on D0 and D28, and the samples were transported to the laboratory within 4 h in cold containers (<10°C) and stored at -80°C until analysis.

During the study, subjects were required to record their defecation frequency, stool consistency (according to the Bristol Stool Form Scale, BS) (Odonnell et al., 1990), and constipation-related symptoms score in a diary (Sakai et al., 2011). The subjects received adequate training before recording their BS and constipation-related symptoms. For the constipation-related symptoms, painful effort a feeling of incomplete evacuation and straining during defecation were assessed on a four-point scale (1, none; 2, little; 3, some; and 4, a lot). The number of minutes spent in the lavatory per attempt was also assessed: less than 5 min was scored as 1, no less than 5 and less than 10 as 2, no less than 10 and less than 20 as 3, and more than 20 as 4. Unsuccessful defecation attempts and abdominal discomfort were recorded in the diary on a daily basis (1, never; 2, rarely; 3, sometimes; and 4, always).

### Measurement of Fecal Water Content and pH

The feces were first thawed at 4°C. Then, the fecal sample (0.5 ± 0.1 g) was moved to a clean plastic tube. The total weight and the net weight of the tube were measured (ALC-201, Acculab) as M1 and M2 (g), respectively. The tube with feces was frozen at -80°C for 2 h and then it was lyophilized by a lyophilizer (LGJ-18, Beijing Songyuan Huaxin Technology Development Co., Ltd.) for 48 h. The tube with the lyophilized feces was weighed as M3. The fecal water content was calculated by the equation: fecal water content = (M1 - M3)/(M1 - M2) × 100%. The fecal pH was measured in duplicate at room temperature by a semi-solid pH detector (Testo 206 pH2, Germany).

### Measurement of Fecal SCFA Levels

The SCFAs were extracted from the stool samples as previously described (Goossens et al., 2003). The levels of acetic acid, propionic acid and butyric acid were quantified by a GC 7890A gas chromatography system (GC, Agilent, Santa Clara, CA, United States) equipped with a flame ionization detector and an HP-FFAP chromatographic column (25 m, 0.32 mm, 0.5 μm) (Agilent, Santa Clara, CA, United States). The program was set as below: maintained at 50°C for 3 min, increased by 5°C/min to 140°C, kept at 140°C for 1 min, increased by 30°C/min to 240°C, and held at 240°C for 3 min. The temperature of the injector was 270°C, and the injection type was splitless. The carrier gas was nitrogen gas, and 1 μL of the supernatant was injected for the GC measurement. The peak area ratio of the target SCFA in the sample compared to that of to internal standard (5 mM heptatonic acid) was measured first, and then it was corrected by a relative correction factor. The corrected peak area ratio was used to calculate the concentration of the SCFA.

### Measurement of Non-volatile Fecal Metabolites by GC/MS

The fecal non-volatile metabolites were extracted and derivatization was performed according to the protocol described previously (Yu et al., 2018). A GC/MS analysis was performed using a 7890A gas chromatograph system (Agilent, United States) coupled with a Pegasus HT time-of-flight mass spectrometer (LECO, United States). The system utilized a DB-5MS capillary column coated with 5% diphenyl cross-linked with 95% dimethylpolysiloxane (30 m × 250 μm inner diameter, 0.25 μm film thickness; J&W Scientific, Folsom, CA, United States). An aliquot of the analyte (1 μL) was injected in splitless mode following the previous procedure (Yu et al., 2018).

Chroma TOF 4.3X software (LECO, United States) and the LECO-Fiehn Rtx5 database (LECO, United States) were used for extracting the raw peak, filtering the data baselines and calibrating the baseline, peak alignment, de-convolution analysis, peak identification and calculations of relative abundance by using the area normalization method (Kind et al., 2009). The RI (retention time index) method was used for the peak identification, and the RI tolerance was set to 5000. The metabolic features detected in <50% of the QC samples were removed (Dunn et al., 2011). For the GC-Quad FiehnLib library, the derivatives were named by increasing number according to the retention index.

First, 477 peaks were detected, and 258 metabolites were left after the interquartile range denoising method. Then, the missing values of the raw data were replaced by half of the minimum value. In addition, the area normalization method was employed in this data analysis. The resulting three-dimensional data involving the peak number, sample name, and normalized peak area were input in the SIMCA14.1 software package (MKS Data Analytics Solutions, Umea, Sweden). Based on the name and relative abundance of the metabolites via the area normalization method (Kind et al., 2009), a principal component analysis (PCA) was performed to illustrate the range of the metabolite profiles. To obtain a higher level of group separation and get a better understanding of the variables responsible for the classifications, supervised orthogonal projections to latent structures-discriminate analysis (OPLS-DA) was also applied. A sevenfold cross validation was used to estimate the robustness and the predictive ability of our model, and a permutation test was used in order to further validate the model. To assess the significantly changed metabolites among the groups, the first principal component of variable importance projection (VIP) was obtained from the OPLS-DA (VIP > 1), followed by Student’s *t*-test (*p* < 0.1).

**FIGURE 2:**
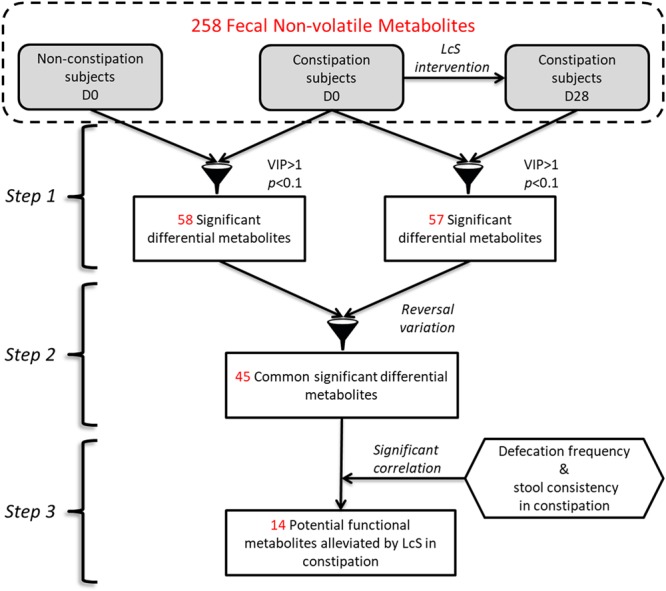
The workflow of determination of potential functional non-volatile fecal metabolites involved in constipation alleviation by LcS.

To determine the fecal metabolites involved in the LcS-induced alleviation of, a workflow was summarized (Figure 2). Briefly, the potential constipation-related metabolites were obtained based on the significant differences in abundance between the constipation and non-constipation groups on D0. Then, the metabolites that changed significantly in constipated patients between D28 and D0 were considered as LcS-regulated metabolites. Correspondingly, the constipation-enriched metabolites that were downregulated by LcS or constipation-reduced metabolites that were upregulated by LcS were considered potential constipation-targeted metabolites regulated by LcS. Then, the relative abundance of these metabolites was correlated with defecation frequency or stool consistency by Pearson correlation analysis in the constipated subjects, and the significance was verified via Student’s *t*-test (*p* < 0.1). The metabolites with a significant correlation with at least one index were considered potential functional metabolites.

### Intestinal Microbiota Analysis by 16S rRNA Sequencing

Bacterial genomic DNA of fecal samples was extracted using the phenol–chloroform method as described previously (Via and Falkinham, 1995). The PCR and amplicon purification and quantification were performed according to the standard procedure (Tang et al., 2018). The purified amplicons were pooled in equimolar concentrations and subjected to paired-end sequencing (2 × 250) on an Illumina MiSeq platform (Illumina, Inc., San Diego, CA, United States). Raw fastq files were demultiplexed and quality-filtered by QIIME (version 1.17), and then the operational taxonomic units (OTUs) were clustered as described in a previous study (Wang J.H. et al., 2017). The OTUs with proportional abundances of at least 0.1% in at least three samples were retained for downstream analysis. The taxonomy of each 16S rRNA gene sequence was analyzed by RDP Classifier^1^ against the silva (SSU115) 16S rRNA database using a confidence threshold of 70% (Amato et al., 2013). The fastq files were available in NCBI Sequence Read Archive (SRA) database (accession no. PRJNA505922).

The relative abundances of the different genera in each sample were calculated and compared between periods and groups using repeated measures ANOVA with RStudio. A principal coordinate analysis (PCoA) based on the weighted UniFrac distances of the OTUs was performed to obtain an overview of the differences in the gut microbes between groups. We also compared the correlation between the target metabolite and the relative abundance of the genera that significantly changed in the constipation group using the above methods.

### Animal Experiments

#### Drug Use

AD tablets (2.5 mg diphenoxylate and 25 μg atropine sulfate monohydrate per tablet) were purchased from Jilin Wantong Pharmacy Group Co. and were used to induce constipation in mice (Xu et al., 2012; Zhu et al., 2016). Pipecolinic acid (PIPA, CAS No. 535-75-1) was purchased from J&K Scientific Ltd. All drugs were suspended in Milli-Q water and fully mixed by vortexing before use.

#### Animals

Eight-week-old male Kunming mice (30–35 g) were purchased from Beijing Vital River Laboratory Animal Technology Co. Ltd. The mice were housed four per cage and maintained in a controlled caged environment (12:12 h light–dark cycle, temperature 22 ± 1°C). All animals were allowed access to a standard diet and tap water *ad libitum*. The animal experiments were carried out in accordance with the recommendations of Animal Management Regulations, Ministry of Science and Technology of the People’s Republic of China. The protocol was approved by the Ethical Committee of Experimental Animal Care of China Agricultural University.

#### Measurement of Small Intestinal Propulsive

A total of 40 mice were randomly divided into five groups (*n* = 8 per group, four mice per cage): normal control group (CON), AD-induced constipation model group (AD group), and AD groups treated with a low (L-PIPA), medium (M-PIPA) or high (H-PIPA) dose of PIPA. After fasting for 16 h with free access to water, the mice in the control group (CON) were administered normal saline, and the other animals were treated with AD (diphenoxylate 5 mg/kg BW; atropine sulfate 0.05 mg/kg BW) by gavage (Xu et al., 2012; Li et al., 2013). Then, 30 min after AD was administered, PIPA was orally consumed by the H-PIPA, M-PIPA and L-PIPA groups (1936, 387, and 77 mg/kg BW, respectively), while an equal volume of normal saline was given to the CON and AD groups. Thirty minutes later, all animals were administered 0.2 mL of 5% (W/V) active charcoal suspension. At 25 min after the charcoal suspension administration, all mice were sacrificed by cervix dislocation. The small intestine (from the pylorus to the caecum) was quickly removed, and its propulsive rate was measured by the ratio of the distance from pylorus to the active charcoal region and the whole length of the small intestine.

#### Numeration of Fecal Pellets

Another 24 mice were randomly divided into three groups (*n* = 8 per group, four mice per cage): normal control group (CON), AD-induced constipation model group (AD), and AD group treated with a high (H-PIPA) dose of PIPA. Normal saline or AD (diphenoxylate 20 mg/kg BW; atropine sulfate 0.2 mg/kg BW; Shan et al., 2010; Qin et al., 2011) were administered to the mice following the procedure described above. One hour later, PIPA was orally given to the H-PIPA group at the same dose as above, while an equal volume of normal saline was given to the AD and CON groups. Fecal pellets were collected and numerated in 11-h period starting at PIPA administration.

#### Measurements of ACh and 5-HT

After the fecal pellet numeration, all mice were immediately sacrificed by cervix dislocation. The colon was carefully dissected and stored at -80°C. The colonic levels of acetylcholine (Ach) and 5-HT were measured using an Ach assay kit and a 5-HT assay kit, respectively, according to the protocol (Nanjing Jiancheng Bioengineering Institute, China). The total protein (TP) was also measured by a BCA protein assay kit (Thermo Scientific), and the concentrations of Ach and 5-HT were expressed in the form of Unit/mg TP.

### Statistical Analysis

In addition to the analysis methods of the non-volatile fecal metabolites and intestinal microbiota in the first trial, a chi-square test was performed for gender, one-way ANOVA was used for age and BMI, a Wilcoxon signed rank test was used to compare the defecation frequency, stool consistency, constipation-related indices, fecal pH, fecal water content and SCFA in same group at different collection times, and a Mann–Whitney test was applied to the same indices as those in the Wilcoxon test to compare them at the same collection time between the constipation and non-constipation groups. In the animal test, a Mann–Whitney test was used to compare the between-group variances. SPSS Version 17.0 (SPSS Inc., Chicago, IL, United States) was used for the chi-square test, one-way ANOVA test, Wilcoxon signed rank test and Mann–Whitney test. The results were presented as the mean ± SD unless otherwise indicated. *p* < 0.05 was considered statistically significant.

## Results

### Effects of LcS on Constipation in Human Subjects

A total of 38 volunteers enrolled and completed the study, including 16 constipated and 22 non-constipated subjects. No significant differences were observed between two groups in terms of gender, age, and body mass index (Table 1).

**Table 1:** Demographical data of subjects.

	Constipation (*n* = 16)	Non-constipation (*n* = 22)	*p*-Value
Gender (% of females)	100	90.9	0.215^a^
Age (year)	35 ± 12	32 ± 10	0.541^b^
BMI (kg/m^2^)	22.43 ± 3.09	21.99 ± 3.47	0.688^b^

*Statistical significance was calculated by ^*a*^ chi-squared test, ^*b*^ one-way ANOVA with LSD test and *p* < 0.05 was considered with significant difference.*

The impact of LcS on defecation (defecation frequency and stool consistency), constipation-related symptoms, fecal characteristics (pH and water content) and fecal SCFA levels are presented in Table 2. Before LcS intake, the constipated subjects had a significantly lower defecation frequency and stool consistency and higher scores of all constipation-related symptoms than non-constipated subjects. Constipated subjects also had lower fecal water contents and butyric acid levels than non-constipated subjects but similar fecal pH and acetic acid and propionic acid levels compared with those of non-constipated subjects (Table 2). Those variables indicated initial differences between the constipation and non-constipation groups. Although 4 weeks of LcS administration did not impact the defecation, fecal characteristics and fecal SCFA levels of non-constipated subjects, LcS significantly improved the defecation frequency and stool consistency and alleviated constipation-related symptoms of constipated subjects (Table 2). The average defecation frequency of constipated subjects significantly increased (from 4.81 to 7.81 times per week, *p* < 0.05) after LcS-beverage intake and gradually reached a similar level to that of non-constipated subjects on D28. LcS administration significantly improved the stool consistency from a dry appearance (2.52 ± 0.70) to a normal appearance (3.68 ± 1.09) in constipated subjects. In addition, LcS supplementation significantly alleviated constipation-related symptoms, and the scores of defecation time, unsuccessful defecation attempts and abdominal discomfort reached same levels as those in non-constipated subjects on D28. However, LcS did not change the fecal pH, water content and SCFA levels in both groups. Compared with the non-constipated subjects, a higher pH and lower butyric acid level were still observed in constipated subjects on D28.

**Table 2:** Effects of LcS on constipation-related indices.

	Constipation			Non-constipation			Constipation vs. non-constipation	
	D0	D28	*p*-Value^a^	D0	D28	*p*-Value^a^	*p*-Value^b^	
							D0	D28
Defecation frequency (times/week)	4.81 ± 1.61	7.81 ± 4.65	**0.004**	15.45 ± 2.79	16.59 ± 4.63	0.343	**<0.001**	**<0.001**
Stool consistency (BS)	2.52 ± 0.70	3.68 ± 1.09	**0.002**	4.18 ± 0.70	3.96 ± 0.60	0.084	**<0.001**	0.171
Pains during defecation	2.03 ± 0.87	1.56 ± 0.68	**0.046**	1.03 ± 0.09	1.12 ± 0.23	0.017	**<0.001**	**0.004**
Incomplete feeling during defecation	2.49 ± 0.65	1.67 ± 0.75	**0.004**	1.18 ± 0.28	1.12 ± 0.22	0.158	**<0.001**	**0.003**
Straining during defecation	2.67 ± 0.65	1.69 ± 0.68	**0.003**	1.21 ± 0.30	1.26 ± 0.33	0.638	**<0.001**	**0.021**
Defecation time	2.05 ± 0.63	1.64 ± 0.60	**0.005**	1.40 ± 0.50	1.46 ± 0.56	0.116	**0.002**	0.246
Unsuccessful defecatory attempts	1.71 ± 0.58	1.23 ± 0.55	**0.002**	1.02 ± 0.06	1.04 ± 0.10	0.336	**<0.001**	0.191
Abdominal discomfort	2.10 ± 0.70	1.34 ± 0.57	**0.002**	1.14 ± 0.26	1.20 ± 0.33	0.286	**<0.001**	0.201
Faecal pH	6.61 ± 0.51	6.63 ± 0.58	0.776	6.37 ± 0.64	6.21 ± 0.47	0.263	0.258	**0.024**
Faecal water %	76.36 ± 7.19	76.07 ± 8.24	0.796	81.93 ± 5.35	81.48 ± 5.93	0.592	**0.009**	0.052
SCFA (mg/g⋅wet feces)				
Acetic acid	30.08 ± 23.27	23.42 ± 12.94	0.234	30.38 ± 10.31	25.79 ± 14.42	0.14	0.372	0.759
Propionic acid	15.32 ± 9.18	14.2 ± 7.28	0.796	18.08 ± 8.10	18.47 ± 8.71	0.685	0.473	0.234
Butyric acid	7.88 ± 5.93	8.81 ± 6.83	0.918	14.71 ± 7.52	13.74 ± 7.56	0.638	**0.004**	**0.017**

*^*a*^Statistical significance was calculated by Wilcoxon signed rank test indicating difference between D0 and D28 in same group, and *p* < 0.05 was considered with significant difference. ^*b*^Statistical significance was calculated by Mann–Whitney *U* test indicating the differences between groups at same time point, and p < 0.05 was considered with significant difference (indicated as bolded values).*

### Effects of LcS on Fecal Non-volatile Metabolites

**FIGURE 3:**
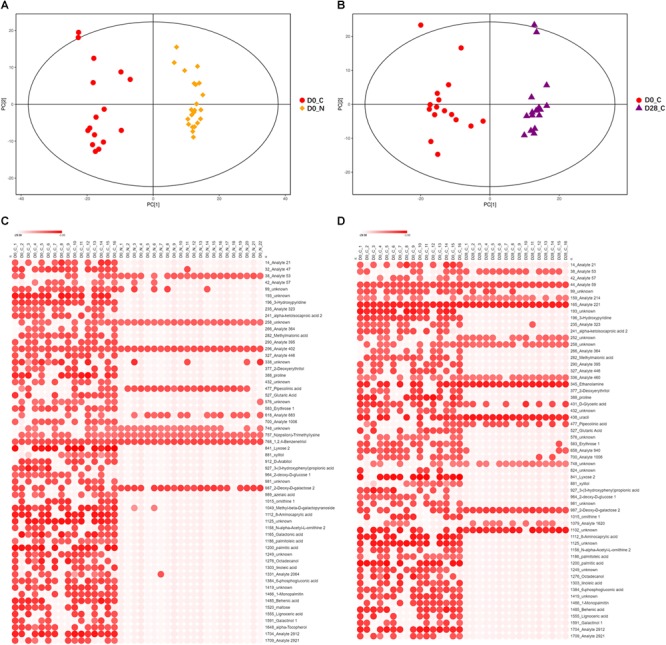
Differences of non-volatile fecal metabolites among subject groups. **(A)** PCA score scatter plot of constipated subjects and non-constipated subjects on D0 (*R*^2^*X* = 0.516, PC1 = 14.00%). **(B)** PCA score scatter plot of constipated subjects before and after LcS intervention (D0 vs. D28, *R*^2^*X* = 0.520, PC1 = 11.90%). **(C)** Heatmap of 58 significantly differential non-volatile fecal metabolites between constipated subjects and non-constipated subjects (VIP > 1 and *p* < 0.1). **(D)** Heatmap of 57 significantly differential non-volatile fecal metabolites in constipated subjects before and after LcS intervention (VIP > 1 and *p* < 0.1). D0_N, non-constipated subjects on D0; D0_C, constipated subjects on D0; D28_C, constipated subjects on D28. In Heatmap, *x*-axis indicated samples and *y*-axis indicated significant differential metabolites. The color scale represented the logarithm value of relative abundance with base 2 of metabolites.

To determine the intestinal metabolites that were involved in LcS-induced alleviation of constipation, fecal non-volatile metabolites were measured in all fecal samples by GC/MS. A total of 258 fecal non-volatile metabolites were obtained and included 55 unknown metabolites, 84 analytes and 119 identity-known metabolites (Supplementary Table 1). The PCA score plot showed that the samples were clustered into two groups, indicating a distinct difference in the metabolite profile between the constipation and non-constipated subjects on D0 (Figure 3A). There were also significant differences in constipation before and after LcS intervention according the PCA (Figure 3B). The OPLS-DA score plot further confirmed the results of the PCA (Supplementary Figures 1A,B). The parameters of the OPLS-DA permutation tests were stable and had a high goodness of fit and prediction (Supplementary Figures 1C,D). The low values of the Q2 intercept indicated the robustness of the models, and thus showed a low risk of over fitting and the reliability of the data obtained.

The statistical analyses showed that 108 metabolites were enriched, while 150 metabolites were reduced in constipated subjects compared with those in the non-constipation group (Supplementary Table 1), and they included 49 significantly increased and 9 significantly reduced metabolites in constipation group according to the parameters of VIP > 1 and *p* < 0.1 (Figure 3C). These 58 significantly different metabolites contained 11 unknown metabolites, 14 analytes and 33 identity-known metabolites.

After LcS intervention, 110 metabolites decreased and 148 increased in the constipation group (Supplementary Table 1). A total of 57 fecal metabolites were significantly affected by LcS (VIP > 1 and *p* < 0.1), including 14 upregulated and 43 downregulated metabolites (Figure 3D). Among these 57 metabolites, 13 belonged to an unknown category, 16 were analytes and 28 were identity-known metabolites.

**FIGURE 4:**
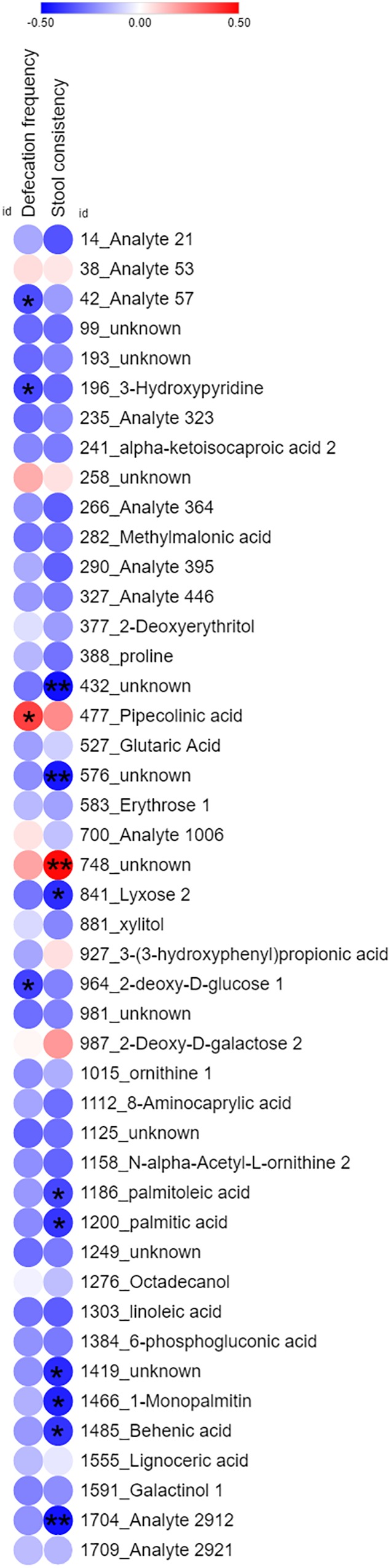
Heatmap of Pearson correlation analysis of 45 common identity-known differential non-volatile fecal metabolites with defecation*(frequency and stool consistency in constipation subjects. The color dots indicated correlation coefficient. The red dots indicated positive correlation, while blue dots indicated negative correlation. ^∗^ indicated significant correlation (Student’s *t*-test, *p* < 0.05). ^∗∗^ indicated significant correlation (Student’s *t*-test, *p* < 0.01).)*

By corresponding the constipation-related metabolites to the LcS-regulated metabolites, we found 45 potential constipation-targeted metabolites that were regulated by LcS. A Pearson correlation analysis was used to analyze the correlation of these 45 metabolites with defecation frequency and stool consistency as two constipation-related phenotypes (Figure 4). A total of 14 metabolites had a significant correlation with defecation frequency or stool consistency, including 12 with negative correlation and 2 with positive correlation. The results showed that the identity-known metabolites including 3-hydroxypyridine and 2-deoxy-D-glucose had negative correlations with defecation frequency. Lyxose, palmitoleic acid, palmitic acid, 1-monopalmitin and behenic acid were negatively correlated with stool consistency. PIPA had a with significant positive correlation (*p* = 0.035) with defecation frequency and was considered as one of potential functional metabolites in LcS-induced constipation alleviation. Retrospectively, the abundance of PIPA significantly enriched in non-constipated subjects than that of constipated patients on D0 (Supplementary Table 1). LcS intervention significantly increased the average level of fecal PIPA in constipated group (Supplementary Tables 1, 2). In addition, the abundance of PIPA did not changed significantly between D28 and D0 in non-constipated subjects (Supplementary Table 1). Therefore, we assumed that LcS could stimulate a noticeable increase in PIPA in the gut after 4 weeks intervention. The anti-constipation function of PIPA was further testified in mice model.

### Effects of LcS on Intestinal Microbiota

To investigate the intestinal bacteria that were related to the functional metabolites in LcS-induced constipation alleviation, the microbiota was analyzed by 16S rRNA sequencing. A total of 4,310,192 filtered high-quality sequence reads were obtained (with a mean of 56,713 reads/sample and an average length of 433 bp). A total of 1,141 OTUs belonging to 425 genera were obtained and the relative abundance of genus was shown in Supplementary Table 3. The stability of the rarefaction curves suggested that most of the diversity had already been covered (Supplementary Figure 2). Constipated patients showed significant lower bacterial diversity than that of non-constipated subjects on D0 based on Shannon, ACE and Chao indices, and these variances were not observed on D28. Intake of LcS decreased bacterial diversity in non-constipated subjects (reduced ACE and Chao indices on D28), but LcS did not affect the bacterial diversity in constipated patients (Supplementary Table 4). As shown in PCoA score plot, distinct differences were observed in the microbiota between the constipated and non-constipated subjects (Supplementary Figure 3A). LcS intervention noticeably altered the microbiota in constipated subjects, which was also found in the PCoA plot (Supplementary Figure 3B). The intake of LcS significantly increased the relative abundances of OTU696 belonging to *Lactobacillus* genus in both constipated and non-constipated subjects (Supplementary Figure 4), but no obvious variance was observed in *Lactobacillus* genus. The Wilcoxon signed rank test showed that the relative abundance of 12 genera were increased or reduced: *Raoultella* and *Halomonas* were reduced, and *Gelria, Syntrophomonas, Hungatella, Proteiniphilum, Staphylococcus, Aminobacterium, Brucella, Desulfosporosinus*, unclassified_p__*Firmicutes* and uncultured_f__Family_XIV were increased, which led to the majority of the variation caused by LcS (Supplementary Table 5). The Pearson correlation analysis indicated a significant positive correlation between *Brucella* and PIPA statistically (Supplementary Figure 5), which suggested that the increased *Brucella* in the gut might have closely relationship with elevated PIPA level (correlation coefficient = 0.668, *p* < 0.001).

### Effects of PIPA on Constipation in Mice

To verify the potential constipation alleviation effects of PIPA involved in LcS intervention, an AD mice model was employed. The small intestine propulsive rate, number of fecal pellets and colonic neurotransmitter concentrations were evaluated in AD mice with PIPA supplementation. Compared to the CON group, the AD group had a significantly lower small intestine propulsive rate (Table 3). PIPA intervention increased the small intestine propulsive rate of the AD mice in a dose-dependent manner. The H-PIPA group showed a significant increase in the small intestine propulsive rate compared with the AD group (from 25.45 ± 10.05 to 39.68 ± 7.96, *p* < 0.05), while the M-PIPA and L-PIPA groups showed a slightly improved small intestine propulsive rate that was significantly different from that of the AD group. Not unexpectedly, the AD group also showed a distinctly lower number of fecal pellets than the control group (CON), and the high dose of PIPA (H-PIPA) significantly increased the number of fecal pellets by 1.88-fold (from 30.38 ± 7.73 to 57.38 ± 9.46, *p* < 0.05) compared with the AD group. The results clearly showed that the high dose of PIPA could improve the intestinal movement and alleviate constipation symptoms in AD-induced constipation mice. To further investigate the possible anti-constipation mechanisms of PIPA, ACh and 5-HT, which are the neurotransmitters related to intestinal tract movement, were measured in the colonic tissue. The results showed that the AD groups had lower levels of ACh and 5-HT in colonic tissue and that the H-PIPA dose induced an increase in both colonic ACh and 5-HT but without significant differences from those in the AD group.

**Table 3:** Effects of PIPA on constipation in mice.

	CON	AD	H-PIPA	M-PIPA	L-PIPA
Small intestine propulsive (%)	54.34 ± 12.55^a^	25.45 ± 10.05^c^	39.68 ± 7.96^b^	32.11 ± 6.79^c^	32.07 ± 7.65^c^
Excretive fecal pellets	79.23 ± 6.14^a^	30.38 ± 7.73^c^	57.38 ± 9.46^b^	∖	∖
Colonic neurotransmitters					
ACh (μg/mg⋅TP)	9.27 ± 15.55	8.24 ± 5.49	11.16 ± 10.37	∖	∖
5-HT (ng/mg⋅TP)	2.35 ± 0.71^a^	1.46 ± 0.67^b^	1.62 ± 0.6^ab^	∖	∖

*^*a,b,c*^ Different letters indicated significant differences among groups (Mann–Whitney *U* test, *p* < 0.05). ∖indicated these tests were not performed in the study.*

## Discussion

In this study, we confirmed the constipation alleviation function of LcS in Chinese adults with constipation. By metabolomics methods, we revealed that PIPA was the potential functional fecal non-volatile metabolite that was responsible for LcS-mediated constipation alleviation. Furthermore, we verified that PIPA could attenuate constipation symptoms in a mouse model and that neurotransmitters may be partially involved in this process. As far as we know, this is the first study to show the specific gut metabolite that is regulated by probiotics to alleviate constipation.

Constipation is characterized by a decrease in colonic motility, which is affected by the gut flora and metabolites derived from them (Quigley and Spiller, 2016). As one of widely used probiotic strains, the anti-constipation function of LcS has been evaluated in different populations, including elderly patients (van den Nieuwboer et al., 2015), women during puerperium (Sakai et al., 2015), patients who have undergone a gastrectomy (Aoki et al., 2014), constipated patients (Koebnick et al., 2003; Schlieger et al., 2006; Mazlyn et al., 2013), patients with end-stage renal disease (Nakabayashi et al., 2011), and healthy populations (Sakai et al., 2011). LcS lead to significant improvements in the defecation frequency, stool consistency, colonic transit time and constipation-related symptoms in patients in previous studies. However, the anti-constipation mechanism of LcS is still unclear.

Several studies suggested that alterations in SCFAs and putrefactive metabolites in the gut may be related to bowel movement and constipation (Lewis and Heaton, 1997; Roager et al., 2016). SCFAs are mainly produced from carbohydrate fermentation in the colon and are necessary to maintain colonic health, including colonic motility (Lewis and Heaton, 1997; Wong et al., 2006), which is attributed to the enteric neurons and secretion of PYY regulated by SCFAs (Soret et al., 2010; Larraufie et al., 2018). The peristalsis reflex could also be triggered by SCFAs via the sequential release of 5-HT from mucosal cells and the activation of 5-HT_4_R on sensory nerve terminals, consequently stimulating gut propulsive motility (Grider and Piland, 2007). However, as reported by Sakai et al. (2011), LcS significantly reduced the incidence of hard or lumpy stools without affecting the levels of acetic acid, propionic acid and butyric acid in healthy subjects. Similar results were also obtained in this study, in which LcS significantly alleviated constipation symptoms in constipated patients but did not impact the level of acetic acid, propionic acid and butyric acid in their gut. These results suggested that SCFAs may not contribute to the bowel movement regulated by LcS. On the other hand, gut bacteria-derived protein-related products including *p*-cresol sulfate and *p*-cresol glucuronide have significant positive associations with colonic transit time (Roager et al., 2016). Their precursor, *p*-cresol, was reduced in the serum of hemodialysis patients after LcS-containing synbiotic intake, which was considered the association to normalization of bowel habits by LcS (Nakabayashi et al., 2011). However, Aoki et al. showed that LcS intake slightly increased the fecal level of *p*-cresol in gastrectomized subjects, indicating that *p*-cresol has an unclear role in LcS-mediated constipation alleviation (Aoki et al., 2014). Due to the complex composition of the gut microbiota and its metabolites, it is difficult to assess the relationship between probiotics and gut metabolites by single metabolite analyses. In this study, we employed metabolomic approaches to investigate the interaction between LcS and metabolites, as well as host constipation symptoms. With GC/MS, we obtained a total of 258 metabolites to further analysis. We first compared the differences between the constipated and non-constipated subjects and obtained possible constipation-related metabolites. Then, we corresponded these metabolites with LcS-regulated metabolites in the constipation group and found potential constipation-alleviating metabolites associated with LcS. We further correlated these potential constipation-alleviating metabolites with primary LcS-ameliorated defecation phenotypes (defecation frequency and stool consistency). With the abovementioned procedures, we identified one identity-known metabolite, PIPA, that had a significant positive correlation with defecation frequency. We also found 748_unknown as a metabolite that was positively correlated with stool consistency (*p* = 0.001); however, we could not identify it in the current MS library for further analysis. Therefore, in this study, PIPA was reasonably assumed to be best possible metabolite that mediated the anti-constipation effects of LcS.

PIPA is considered to contain one amino acid, and its levo isomer called pipecolic acid is involved in epilepsy (Plecko et al., 2005). Until now, PIPA has rarely been studied as a gut metabolite, and its relationship to the host has been rarely studied as well. A recent study reported that orally taking L-lysine, which is a precursor of PIPA, could improve the small intestine propulsive rate in mice (Nakato et al., 2017). Although it seems that PIPA may be involved in intestinal motility, there is not an abundance of data or direct evidence to support this hypothesis. In this study, PIPA was identified as a non-volatile fecal metabolite by GC/MS, and its important impacts on constipation were also discovered in the adult gut. Compared to non-constipated subjects, constipated subjects had a significantly lower abundance of PIPA in their feces, suggesting its close relationship with constipation. In addition, LcS intake increased the abundance of PIPA in constipated subjects, and PIPA was also positively correlated to defecation frequency, indicating its role in constipation alleviation via LcS. Furthermore, PIPA successfully increased the small intestine propulsive rate and the number of fecal pellets in a dose-dependent manner in the AD-induced constipation mouse model. The median PIPA dosages of gavage were calculated according to 10 times of the average relative abundance of PIPA in constipated subjects at D28 (0.049%), the daily fecal weight (about 2 g), and body weight of mice (about 25 g). Previous study showed that average relative abundance of PIPA in feces from male mice was about 0.194% (Snijders et al., 2017). The concentration of H-PIPA (2.45%) and M-PIPA (0.49%) used in this study were much higher than that of normal male mice. Furthermore, the effects of PIPA on constipation exhibited a clear dose-effect relationship. Although without the data of fecal PIPA concentration in mice, the results credibly confirmed its significant role in constipation alleviation. Consequently, we tried to reveal the mechanisms of PIPA in constipation alleviation. Given that the function of enteric neurons is important to constipation, we analyzed the level of neurotransmitters, 5-HT and ACh, in colonic tissues, which are related to colonic motility and are impacted by indigenous intestinal microbiota (Kendig and Grider, 2015; Yano et al., 2015). The results showed PIPA slightly increased 5-HT and Ach levels, which were suppressed by AD. This result suggested 5-HT and Ach may be partly involved in PIPA-mediated constipation alleviation, but the distinct mechanism should be defined in future. In addition, the microbiota analysis combined with the correlation analysis showed that the abundance the *Brucella* genus in the gut had a statistical correlation with the level of PIPA. As *Brucella* is a zoonotic pathogen, little literature has referred to the metabolism of PIPA in *Brucella*. Gut bacteria harbor complex metabolic pathways, and ubiquitous cross-feeding network may lead to multiple microorganisms participating in PIPA metabolism. The interaction between PIPA and the microbiota should be assessed in the future.

## Conclusion

*Lactobacillus casei* strain Shirota intake increased defecation frequency, normalized stool consistency, and improved constipation related symptoms in Chinese adults with constipation. Through the use of metabolomic approaches, PIPA, which is a non-volatile metabolite in the gut, was identified as a potential target mediator in LcS-mediated constipation alleviation. Furthermore, PIPA could improve constipation symptoms, as shown by increases in the small intestine propulsive rate and the number of fecal pellets in a constipation mouse model, raising its possibility in the constipation therapy. Future studies should verify the anti-constipation function of PIPA and elucidate its potential mechanisms.

## Data Availability

Publicly available datasets were analyzed in this study. This data can be found here: https://www.ncbi.nlm.nih.gov/bioproject/PRJNA505922.

## Author Contributions

YO, SC, FR, and LZ designed the study and revised the manuscript. YO and SC performed the most experiments. MZ, SG, HG, and HZ contributed to the human study. YO, SC, and LZ analyzed the data. YO wrote the manuscript.

## Conflict of Interest Statement

The authors declare that the research was conducted in the absence of any commercial or financial relationships that could be construed as a potential conflict of interest.

## Footnotes

1 http://rdp.cme.msu.edu/
