# Supplementary material for: Lactobacillus casei Strain Shirota Alleviates Constipation in Adults by Increasing the Pipecolinic Acid Level in the Gut
Source: Front Microbiol. 2019 Feb 21;10:324. doi: 10.3389/fmicb.2019.00324 (PMC6394200; doi:10.3389/fmicb.2019.00324)
Supplement: Supplementary file 1 [file Data_Sheet_1.PDF]

Supplementary Figures

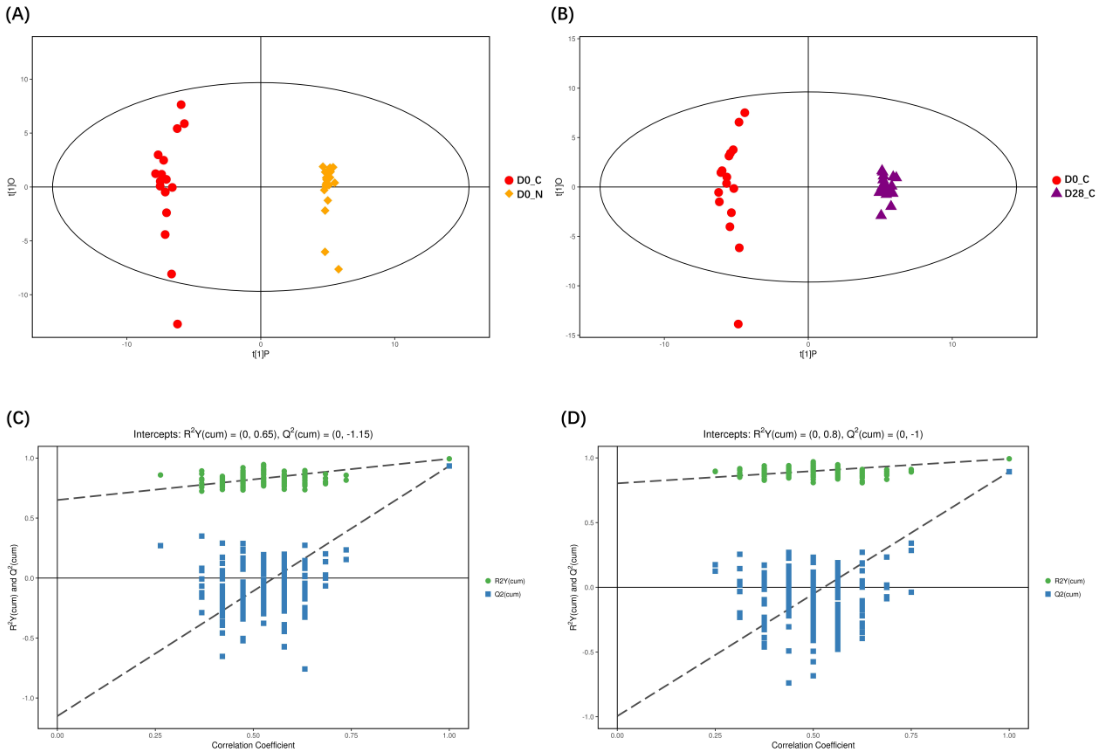

Supplementary Figure 1. OPLS-DA score scatter plot and OPLS-DA permutation test of non-volatile fecal metabolites among subject groups. **(A)** OPLS-DA score scatter plot of constipated subjects and non-constipated subjects on D0 (R^2X=0.205, O1=6.56%). **(B)** OPLS-DA score scatter plot of constipated subjects before and after LcS intervention (R^2X=0.182, O1=6.28%). **(C)** OPLS-DA permutation test of constipated subjects and non-constipated subjects on D0 (R^2Y=0.994, Q^2=0.934). **(D)** OPLS-DA score scatter plot of constipated subjects before and after LcS intervention (R^2Y=0.993, Q^2=0.893). D0_N: non-constipated subjects on D0. D0_C: constipated subjects on D0. D28_C: constipated subjects on D28.

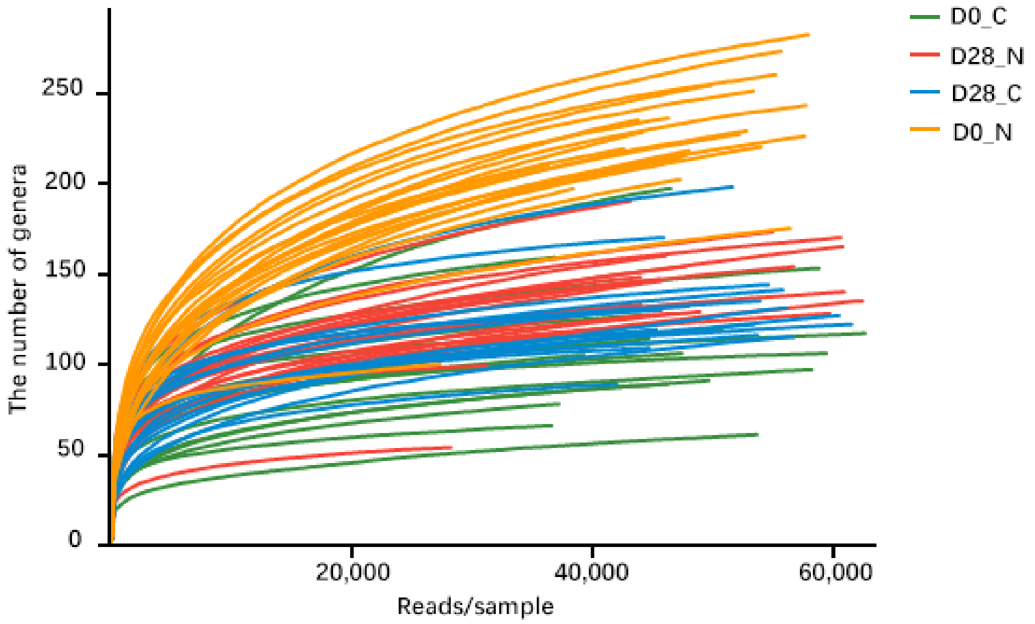

Supplementary Figure 2. Rarefaction curves of fecal samples in genus level. D0_N: non-constipated subjects on D0. D0_C: constipated subjects on D0. D28_N: non-constipated subjects on D28. D28_C: constipated subjects on D28.

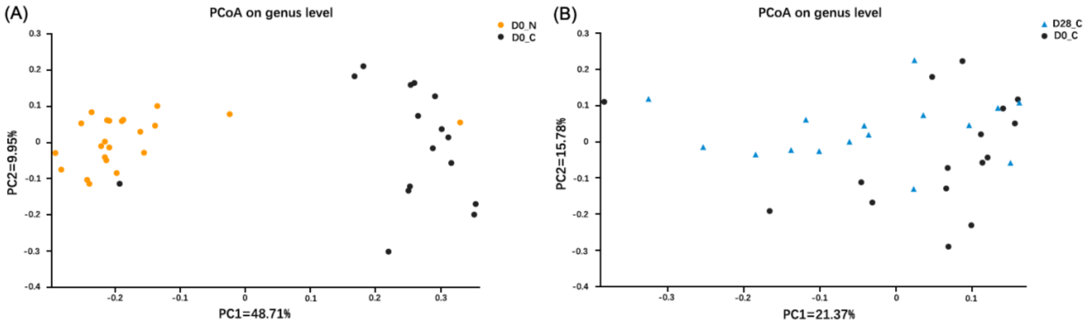

Supplementary Figure 3. PCoA plots of intestinal microbiota at genus level. **(A)** PCoA score scatter plot of constipated subjects and non-constipated subjects on D0. **(B)** PCoA score scatter plot of constipated subjects before and after LcS intervention (D0 vs D28). D0_N: non-constipated subjects on D0. D0_C: constipated subjects on D0. D28_C: constipated subjects on D28.

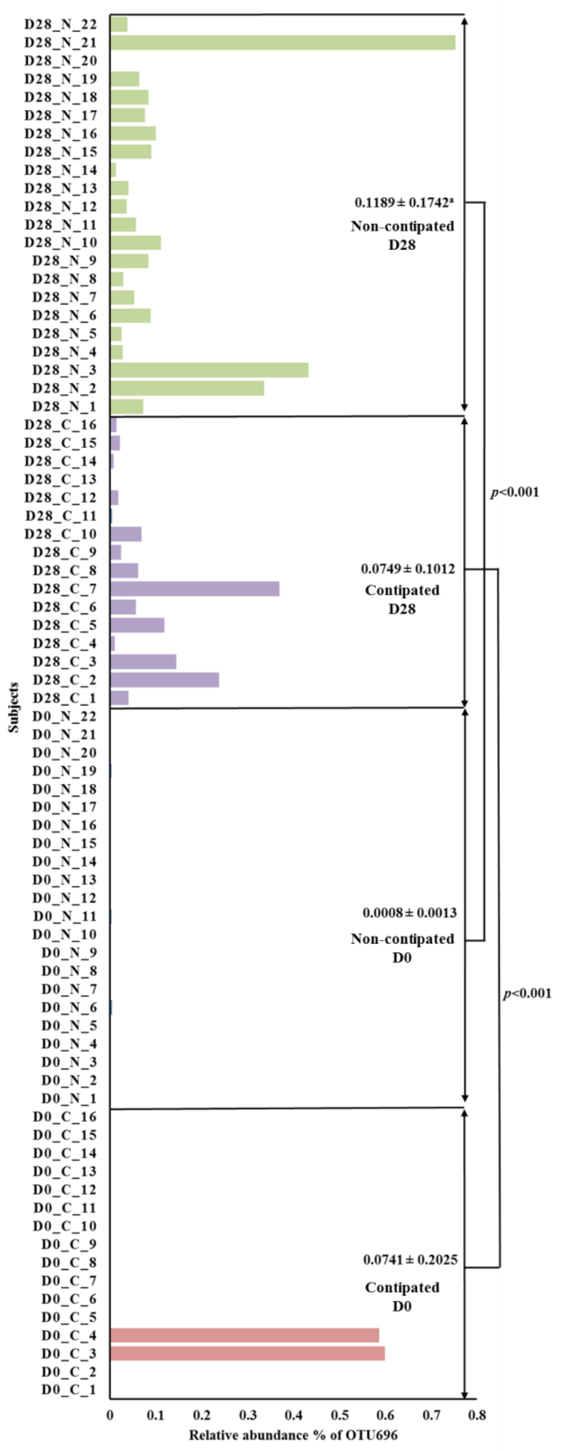

Supplementary Figure 4. The relative abundance % of OTU696 belonging to *Lactobacillus* genus. D0_C: constipated subjects on D0. D28_C: constipated subjects on D28. D0_N: non-constipated subjects on D0. D28_N: non-constipated subjects on D28. [a] indicated the mean and standard deviation of the group that double arrow showed. Wilcoxon rank-sum test were used on statistical analysis and $p<0.05$ was considered significant.

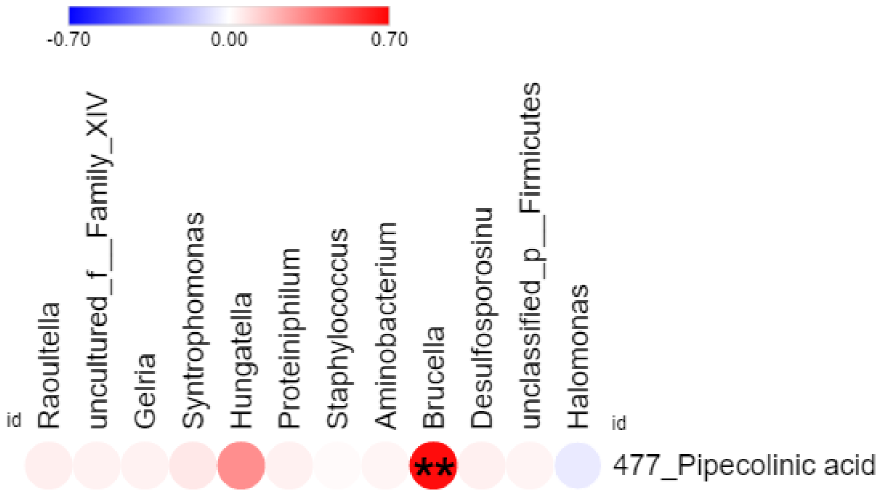

Supplementary Figure 5. Heatmap of Pearson correlation of PIPA with LcS-regulated bacteria genera in constipation subjects. The color dots indicated correlation coefficient. The red dots indicated positive correlation, while blue dots indicated negative correlation. * indicated significant correlation (Student's t test, $p < 0.05$).
